# Validation of a novel multiplex real-time PCR assay for *Trypanosoma cruzi* detection and quantification in açai pulp

**DOI:** 10.1371/journal.pone.0246435

**Published:** 2021-02-02

**Authors:** Paula Finamore-Araujo, Amanda Faier-Pereira, Carlos Ramon do Nascimento Brito, Eldrinei Gomes Peres, Klenicy Kazumy de Lima Yamaguchi, Renata Trotta Barroso Ferreira, Otacilio Cruz Moreira

**Affiliations:** 1 Plataforma Fiocruz de PCR em Tempo Real RPT09A –Laboratório de Biologia Molecular e Doenças Endêmicas, Instituto Oswaldo Cruz, Fundação Oswaldo Cruz, Rio de Janeiro, Brazil; 2 Departamento de Análises Clínicas e Toxicológicas, Centro de Ciências da Saúde, Universidade Federal do Rio Grande do Norte, Natal, RN, Brazil; 3 Departamento de Química, Universidade Federal do Amazonas, Manaus, AM, Brazil; 4 Instituto de Saúde e Biotecnologia, Universidade Federal do Amazonas–Campus Coari, Amazonas, Brazil; 5 Instituto Nacional de Controle de Qualidade em Saúde, Fundação Oswaldo Cruz, Rio de Janeiro, Brazil; Instituto Rene Rachou, BRAZIL

## Abstract

In Brazil, orally acquired *T*. *cruzi* infection has become the most relevant transmission mechanisms from public health perspective. Around 70% of new Chagas disease cases have been associated with consumption of contaminated food or beverages. Açai (*Euterpe oleracea* and *Euterpe precatoria*) is currently one of the most commercialized Amazonian fruits in the Brazilian and international markets. Therefore, it has become important to incorporate in the production process some procedures to measure out effective hygiene and product quality control required by global market. Molecular methods have been developed for rapid detection and quantification of *T*. *cruzi* DNA in several biological samples, including food matrices, for epidemiological investigation of Chagas disease and food quality control. However, a high-performance molecular methodology since DNA extraction until detection and quantification of *T*. *cruzi* DNA in açai berry pulp is still needed. Herein, a simple DNA extraction methodology was standardized from the supernatant of açai berry pulp stabilized in a 6M Guanidine-HCl/0.2M EDTA buffer. In addition, a multiplex real time qPCR assay, targeting *T*. *cruzi* DNA and an Exogenous Internal Positive Control was developed and validated, using reference from all *T*. *cruzi* DTUs and commercial samples of açai pulp, from an endemic municipality with previous history of oral Chagas disease outbreak. Thus, a high-sensitivity qPCR assay, that could detect up to 0.01 parasite equivalents/mL in açai, was reached. As of the 45 commercial samples analyzed, 9 (20%) were positive for *T*. *cruzi*. This high-sensitive, fast, and easy-to-use molecular assay is compatible with most of the laboratories involved in the investigations of oral Chagas disease outbreaks, representing an important tool to the epidemiology, control, and surveillance of Chagas disease.

## Introduction

Chagas disease is a neglected tropical illness, caused by the flagellated and heteroxenous protozoan *Trypanosoma cruzi*. Although it is considered endemic in 21 countries in America, mainly affecting Latin America, Chagas disease has now spread to previously non-endemic areas, due to the increased population migration between Latin America and the rest of the world [1]. As of today, it is estimated that six to seven million people are infected by *T*. *cruzi*, mostly in the endemic areas, and approximately 75 million people are at risk of infection [2]. *T*. *cruzi* is represented by a set of sub-populations, comprising isolates and strains, which alternates between mammalian hosts and insect vectors, with high genetic variability and notable heterogeneity of clinical behavior and parasite-host relationship. Currently, seven genotypes, or Discrete Typing Units (DTUs) are recognized: TcI-TcVI, and Tcbat, this being last reported as TcVII [3, 4].

While Chagas disease is still often associated as a vector borne disease, its transmission can occur in other different routes besides vectorial such as blood transfusions, organs transplantation, congenital, laboratory accidents and oral transmission [5]. However, foodborne outbreaks of Chagas disease seem to be importantly increasing through Latin America [6]. Oral transmission can occur due to the consumption of complete triatomines or its feces, containing metacyclic trypomastigotes, which is inadvertently processed with food, especially fruits preparations [7–9].

In Brazil, the oral route of *T*. *cruzi* infection has become one of the most relevant transmission mechanisms from the public health perspective. Between the years 2000 and 2011, 1252 cases of acute Chagas disease were reported and, in these, 70% have been associated with consumption of contaminated food or beverages [6, 10]. Some of these orally acquired Chagas Disease are related to the consumption of fresh foods or drinks, as sugar cane juice, açai berry (*Euterpe oleracea*) and bacaba wine (*Oenocarpus bacaba*) [6, 11]. Among the Brazilian outbreaks, it is worth mentioning a suspected *T*. *cruzi* oral transmission through açai juice in the Northern Brazil, where cases of this transmission route show signs of increase. The outbreak occurred in 2008 at Coari city, in the interior of the Amazonas state, where 25 people got infected by *T*. *cruzi* [12]. Other countries around Latin America, suchlike Argentina, Bolivia, Colombia, Ecuador, and Venezuela, also have reported multiple Chagas disease outbreaks acquired orally [11]. The largest outbreak described so far occurred in Venezuela, in 2007, affecting 103 people, adults and children from a school in Caracas, that got infected by *T*. *cruzi* after the consumption of contaminated guava juice [13].

The açaizeiro is one of the most socio-economically important palm trees that occurs especially throughout the Amazon region and is particularly abundant in the Eastern Amazon. Three species are popularly known as açai, *Euterpe edulis*, *E*. *oleraceae and E*. *precatoria* [14], however, only the last two have agro-industrial interest. Both species have been shown antioxidant and anti-inflammatory properties with bioactive compounds as anthocyanins, flavonoids and phenolics, more than other fruits, such as grapes, blackberries, blueberries, and strawberries [15, 16]. Most of the açai is derived from the extractive activity, which is the main source of income for the riverine population of the Amazon Basin. In the North of Brazil, mainly, most of the production is still consumed by the local population, in which açai is traditionally ingested "*in natura*", especially in Para and amazon states, the biggest producers and consumers of açai around the world [17]. Furthermore, in the last ten years, there has been an important economic increase, nationally and internationally, in açai-based drinks commercialization. Açai is currently one of the most commercialized Amazonian fruits, not only in the Brazilian market, but also at an international level [18]. Even so, the national and international markets still have potential for considerable expansion of açai commercialization. Therefore, it has become important to incorporate in the production process some procedures to measure out effective hygiene and product quality control required by global market [19, 20].

Until now, the evaluation of the potential for oral transmission of Chagas disease through the consumption of açai-based products is determined by clinical or parasitological investigations and are based on traditional methods, such as culture and microscopic observations [17]. Molecular methods based on PCR have been developed for the rapid detection and quantification of *T*. *cruzi* DNA in several biological samples [21]. More recently, it has also begun to be used to test food matrices, as a powerful tool in the epidemiological investigation of Chagas disease [22–26]. Moreover, the use of PCR for the detection of *T*. *cruzi* DNA has already been described in literature, since several studies demonstrate that this methodology presents greater sensitivity and specificity in the face of conventional parasitological methods [21]. Nevertheless, it is still demanded the development of methodologies capable to detect *T*. *cruzi* in food samples and, thereby, plan strategies to get a better understanding of oral transmission outbreaks and to assure the quality of the products thar are being commercialized.

## Results

**Fig 1:**
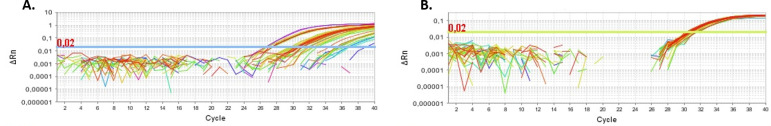
Representative amplification plots, targeting *T*. *cruzi* nuclear satellite DNA (A) and EXO-IPC DNA (B). DNA samples were extracted from GEA spiked with EXO-IPC synthetic DNA, using a silica-column based commercial kit.

In this study, an efficient DNA extraction method for açai pulp samples stabilized in guanidine-EDTA solution, in conjunction with a real-time PCR assay to rapidly assess, with reproducible protocols, the presence of *T*. *cruzi* DNA in açai pulp samples, were developed and validated. To increase the sensitivity of the methodology, artificially contaminated açai samples were submitted to a pre-lysis stage followed by a centrifugation step as described in material and methods section. To monitor the entire procedure concerning the stability of DNA and its loss during sample processing, a normalized amount of an exogenous internal positive control DNA (EXO-IPC DNA) was added to each GEA prior DNA extraction. Then, through qPCR assays, EXO-IPC DNA amplification from GEA samples with low *T*. *cruzi* concentrations was assessed (Fig 1).

To evaluate the analytical sensitivity for the *T*. *cruzi* detection in the multiplex assay, GEA samples were spiked with *T*. *cruzi* and serially diluted to reach from 10 to 0.01 parasite equivalents/mL, prior the supernatant isolation and DNA extraction. In addition, 300 μL supernatant aliquots were spiked with EXO-IPC DNA, and satDNA and Exo-IPC amplifications were monitored trough the Ct values at the multiplex real time PCR assay. As shown in Table 1, it is possible to observe the satDNA amplification in all concentrations tested, with Ct values from 27.32±0.34 to 36.82±1.3, reaching a Limit of Detection of 0.01 *T*. *cruzi* equivalents/mL. Furthermore, the Ct values for the Exo-IPC varies only from 30.50±0.12 to 30.92±0.44, regardless the *T*. *cruzi* concentration at the samples, in the range tested.

**Table 1:** Analytical sensitivity for the satDNA detection in GEA samples spiked with *T*. *cruzi* and Exo-IPC DNA. Assay was tested with genomic DNA extracted from GEA samples with different *T*. *cruzi* (Dm28c) concentrations, ranging from 10 to 0.01 *T*. *cruzi* equivalents/mL. All GEA supernatant samples were spiked with 2 μL EXO-IPC DNA prior DNA extraction. Results are shown as Ct mean ± SD for satDNA and Exo-IPC at the multiplex real time PCR assay.

Targets	*T*. *cruzi* equivalents/mL						
	10	1	0.75	0.5	0.25	0.1	0.01
**satDNA**	27.32 ± 0.34	30.22 ± 0.44	30.52 ± 0.31	31.34 ± 0.79	32.14 ± 0.77	35.47 ± 0.68	36.82 ± 1.30
**Exo-IPC**	30.57 ± 0.17	30.59 ± 0.24	30.92 ± 0.44	30.84 ± 0.16	30.50 ± 0.12	31. 10 ± 0.39	30.84 ± 0.47

**Fig 2:**
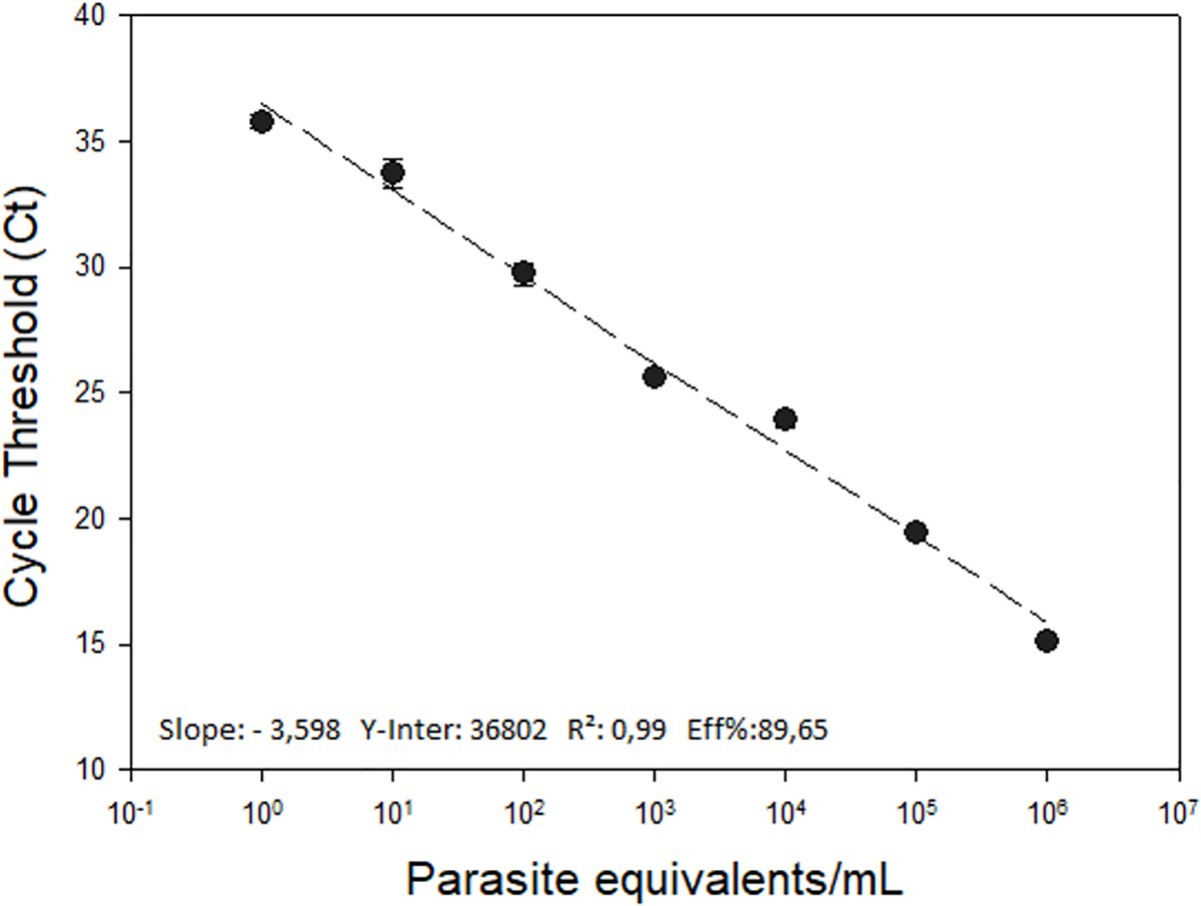
Reportable range for the quantification of *T*. *cruzi* in açai samples by real-time qPCR. A ten-fold serial dilution of DNA extracted from GEA spiked with *T*. *cruzi* was used to generate the standard curve for sat-DNA target, ranging from 10^6^ to 1 Par. Eq./mL. The bottom of the graphic shows the standard curve parameters of the assay.

Following the analytical validation, the reportable range for the *T*. *cruzi* load quantification in açai pulp samples was determined. It was possible to observe that the *T*. *cruzi* DNA detection presented an improved linearity (r^2^ = 0.99) in the range from 10^6^ to 1 parasite equivalents/mL, to the serial dilution of DNA extracted from GEA spiked with *T*. *cruzi* epimastigotes in DNA from negative GEA, as described in the Methods section (Fig 2). Under these conditions, it was possible to obtain a PCR efficiency of 89.65% to the amplification of the satDNA target.

Thereafter, inclusivity and exclusivity assays were performed. For the inclusivity assay. Table 2 shows that the multiplex qPCR could detect all the six *T*. *cruzi* DTUs, from 10^4^ to 0.1 Par. Eq./mL, with Ct values varying from 20.37±1.25 (to TcV, at 10^4^ Par. Eq/mL) to 42.80 (to TcVI, at 0.1 Par. Eq/mL).

**Table 2:** Inclusivity assay for *T*. *cruzi* DTUs. Assay was tested with genomic DNA obtained from a panel of GEA samples spiked with *T*. *cruzi* from six different DTUs. DNA concentrations ranged from 10^4^ to 10^−1^ parasites equivalents/mL. Results are shown as Ct mean ± SD obtained from duplicates of each DNA concentration.

DTUs	10⁴ par.Eq/mL	10^3^ par.Eq/mL	10^2^ par.Eq/mL	10 par.Eq/mL	1 par.Eq/mL	0.1 par.Eq/mL
	Ct ± SD	Ct ± SD	Ct ± SD	Ct ± SD	Ct ± SD	Ct ± SD
**TcI (Dm28c)**	24.60 ± 0.61	29.67 ± 2.03	32.25 ± 0.16	34.32 ± 1.59	37.50 ± 0.17	40.99 ± 1.82
**TcII (Y)**	20.39 ± 0.02	24.35 ± 0.02	28.44 ± 0.09	30.50 ± 0.51	35.57 ± 0.80	37.28 ± 0.44
**TcIII (3663)**	23.00 ± 1.29	26.79 ± 0.50	32.94 ± 2.80	34.77 ± 1.30	41.18 ± 3.19	39.39 ± 1.26
**TcIV (4167)**	22.57 ± 0.40	27.45 ± 0.30	31.71 ± 0.26	34.42 ± 0.36	38.00 ± 5.13	39.58
**TcV (LL014)**	20.37 ± 1.25	24.14 ± 0.27	28.27 ± 0.20	30.51 ± 0.62	34.71 ± 1.22	36.74 ± 2.00
**TcVI (CL Brener)**	20.50 ± 0.05	25.20 ± 0.62	28.34 ± 0.73	30.96 ± 0.45	34.67 ± 0.25	42.80*

*The amplification occurred for one technical replicate only.

Regarding the exclusivity assay, it is possible to observe in Table 3 that DNA of other trypanosomatids, as *Leishmania (L*.*) amazonensis*, *L (V*.*) braziliensis*, *Crithidia* sp. and *Herpetomonas* sp., showed no amplification in any concentration tested. However, qPCR was positive for all concentration of DNA extracted from GEA spiked with *T*. *rangeli*, from 10^4^ parasites equivalents/mL to 0.1 par. Eq./mL.

**Table 3:** Exclusivity assay with other Trypanosomatids. Assay was tested with serial dilutions of purified DNAs from different species of Trypanosomatids that ranged from 10^4^ to 0.1 parasites equivalents/mL. Results are shown as Ct mean ± SD obtained from duplicates of each DNA concentration.

Samples	10⁴ par.Eq/mL	10^3^ par.Eq/mL	10^2^ par.Eq/mL	10 par.Eq/mL	1 par.Eq/mL	0.1 par.Eq/mL
	Ct ± SD	Ct ± SD	Ct ± SD	Ct ± SD	Ct ± SD	Ct ± SD
***L*. *amazonensis***	NA	NA	NA	NA	NA	NA
***L*. *braziliensis***	NA	NA	NA	NA	NA	NA
***Crithidia* sp.**	NA	NA	NA	NA	NA	NA
***Herpetomonas* sp.**	NA	NA	NA	NA	NA	NA
***T*. *rangeli***	22.95 ± 0.58	27.83 ± 0.62	31.18 ± 0.30	32.95 ± 0.20	33.94 ± 0.64	38.91 ± 0.60

NA: No amplification

The last step of this study was to perform the validation of the multiplex qPCR assay with field samples. Therefore, 45 samples of açai pulp were collected from different street markets in the city of Coari (Amazonas State, Brazil) and investigated for the presence of *T*. *cruzi* by direct observation at an optical microscope and by the qPCR assay. No *T*. *cruzi* could be detected by microscopy in the açai pulp samples. Afterwards, DNA was extracted from the 45 açai field samples, following the standardized methodology. The mean DNA concentration (± standard deviation) was 86.2±54.3 ng/μL, with minimal and maximal concentration been 36.9 and 347.1 ng/μL, respectively. Regarding the DNA purity, the mean 260/280 and 260/230 nm ratios were 1.8±0.1 and 2.1±0.5, respectively (**S1 Table**). It was possible to detect and quantify *T*. *cruzi* in 9 samples (20%) by the multiplex qPCR, with parasite loads ranging from 0.002 to 19,05 par. Eq./mL, as shown in Table 4. However, 6 from the 9 positive samples presented parasite load below the reportable range (10^6^ to 1 par. Eq./mL). In addition, all samples amplified the Exo-IPC target, with Cts from 28.51 ± 0.75 to 34.94 ± 0.48, validating the true-negative samples.

**Table 4:** Validation of the multiplex real time qPCR assay. Forty-five açai pulp samples were obtained from different points of sale at the Coari city (Amazonas State, Brazil). *T*. *cruzi* was detected and quantified using primers targeting satDNA in the multiplex qPCR assay. Results are indicated as Ct mean ± SD and parasite load is shown as parasite equivalents/mL.

Açai Samples	Targets	Ct ± SD	Parasite Load (Par.Eq/mL)^&^	Microscopy
**1**	satDNA	36.42	19.05	Negative
	Exo-IPC	33.17 ± 0.39		
**2**	satDNA	34.64	0.21*	Negative
	Exo-IPC	33.23 ± 0.66		
**3**	satDNA	35.97	0.10*	Negative
	Exo-IPC	31.24 ± 2.56		
**4**	satDNA	41.99	0.002*	Negative
	Exo-IPC	34.94 ± 0.48		
**5**	satDNA	37.49	10.60	Negative
	Exo-IPC	34.27 ± 0.12		
**6**	satDNA	37.91	8.44	Negative
	Exo-IPC	32.84 ± 0.06		
**7**	satDNA	39.31 ± 1.90	0.02 ± 0.01*	Negative
	Exo-IPC	33.40 ± 0.47		
**8**	satDNA	37.67	0.03*	Negative
	Exo-IPC	28.53 ± 0.42		
**9**	satDNA	37.29	0.04*	Negative
	Exo-IPC	29.88 ± 0.02		
**10**	satDNA	NA	Negative	Negative
	Exo-IPC	32.20 ± 0.42		
**11**	satDNA	NA	Negative	Negative
	Exo-IPC	34.80 ± 0.94		
**12**	satDNA	NA	Negative	Negative
	Exo-IPC	33.09 ± 0.21		
**13**	satDNA	NA	Negative	Negative
	Exo-IPC	33.99 ± 0.08		
**14**	satDNA	NA	Negative	Negative
	Exo-IPC	32.54 ± 0.07		
**15**	satDNA	NA	Negative	Negative
	Exo-IPC	32.82 ± 0.12		
**16**	satDNA	NA	Negative	Negative
	Exo-IPC	33.11 ± 0.08		
**17**	satDNA	NA	Negative	Negative
	Exo-IPC	33.02 ± 0.28		
**18**	satDNA	NA	Negative	Negative
	Exo-IPC	32.88 ± 0.37		
**19**	satDNA	NA	Negative	Negative
	Exo-IPC	32.54 ± 0.41		
**20**	satDNA	NA	Negative	Negative
	Exo-IPC	32.72 ± 0.15		
**21**	satDNA	NA	Negative	Negative
	Exo-IPC	32.23 ± 0.73		
**22**	satDNA	NA	Negative	Negative
	Exo-IPC	31.89 ± 0.25		
**23**	satDNA	NA	Negative	Negative
	Exo-IPC	31.61 ± 0.25		
**24**	satDNA	NA	Negative	Negative
	Exo-IPC	31.92 ± 0.27		
**25**	satDNA	NA	Negative	Negative
	Exo-IPC	32.90 ± 0.19		
**26**	satDNA	NA	Negative	Negative
	Exo-IPC	32.18 ± 0.16		
**27**	satDNA	NA	Negative	Negative
	Exo-IPC	32.63 ± 0.16		
**28**	satDNA	NA	Negative	Negative
	Exo-IPC	32.62 ± 0.90		
**29**	satDNA	NA	Negative	Negative
	Exo-IPC	31.78 ± 0.16		
**30**	satDNA	NA	Negative	Negative
	Exo-IPC	33.42 ± 0.21		
**31**	satDNA	NA	Negative	Negative
	Exo-IPC	33.07 ± 0.05		
**32**	satDNA	NA	Negative	Negative
	Exo-IPC	34.10 ± 0.26		
**33**	satDNA	NA	Negative	Negative
	Exo-IPC	33.39 ± 0.02		
**34**	satDNA	NA	Negative	Negative
	Exo-IPC	32.94 ± 0.16		
**35**	satDNA	NA	Negative	Negative
	Exo-IPC	31.54 ± 0.86		
**36**	satDNA	NA	Negative	Negative
	Exo-IPC	32.73 ± 0.31		
**37**	satDNA	NA	Negative	Negative
	Exo-IPC	29.41 ± 0.24		
**38**	satDNA	NA	Negative	Negative
	Exo-IPC	29.20 ± 0.60		
**39**	satDNA	NA	Negative	Negative
	Exo-IPC	29.63 ± 0.45		
**40**	satDNA	NA	Negative	Negative
	Exo-IPC	28.51 ± 0.75		
**41**	satDNA	NA	Negative	Negative
	Exo-IPC	29.23 ± 0.11		
**42**	satDNA	NA	Negative	Negative
	Exo-IPC	28.68 ± 0.01		
**43**	satDNA	NA	Negative	Negative
	Exo-IPC	30.20 ± 0.19		
**44**	satDNA	NA	Negative	Negative
	Exo-IPC	29.87 ± 0.40		
**45**	satDNA	NA	Negative	Negative
	Exo-IPC	34.08 ± 0.52		

ND: Non-detected. Sample did not show any Ct value to this target, to the threshold set at 0.02.

*Parasite Load is below the reportable range (10^6^ to 1 par. Eq./mL). The sample is positive for *T*. *cruzi* detection, but the Parasite Load is not quantifiable with accuracy.

^&^Samples without standard deviation for the parasite load amplified in only one technical replicate, at the qPCR assay targeting *T*. *cruzi* satDNA.

## Discussion

In the early 1990s, there was a milestone in the control of Chagas disease in South America. Countries in the Southern Cone (Argentina, Bolivia, Brazil, Chile, Paraguay, and Uruguay) adopted measures to control the vector and to screen and test blood banks [27]. The implementation of programs for the triatomine elimination in Latin American countries has resulted in the control of the transmission in several endemic areas by its main vector, and a significant decrease in the incidence of new cases [1, 28]. Nevertheless, the possibility of vector transmission still prevails since *T*. *cruzi* circulates among other triatomines species and several small mammals in sylvatic environments in which there is human activity [4, 6]. In addition, several outbreaks of oral infection in endemic areas are generally related to the consumption of contaminated [29].

*T*. *cruzi* transmission via the oral route is not a recent event since it represents the main route of contamination between vectors and animals and is one of the main mechanisms of parasite dispersion among mammals [4]. Originally sporadic reported, orally acquired Chagas disease seem to be increasing among populations in several Brazilian states [6] and in other endemic Latin American countries as well [11]. However, in the Northern region of Brazil, the oral transmission poses significant importance, mainly due to the daily diet based on the consumption of food *in natura*, which means unpasteurized homemade or artisan fresh food. Besides that, açai, which is macerated to produce a paste or drink, has been identified as the most frequent food involved in cases of orally acquired acute Chagas disease in Brazil [30, 31].

*T*. *cruzi* oral transmission prevention has proved to be relatively difficult, giving rise to the public health system a new demand to face orally acquired Chagas disease. Although guidelines for minimizing contamination by microorganisms and parasites during the processing in the food chain have already been established [20, 32], it is necessary to assess the quality of açai products that are sold and consumed by the population. Some studies that allow the molecular identification of the parasite in different food sources are already being developed and published [22–26, 30]. Table 5 resumes all the recent manuscripts reporting the use of molecular methods to detect *T*. *cruzi* in açai samples in comparison to the present study. It is possible to observe important differences between them, especially regarding DNA extraction methodologies and the use of internal controls.

**Table 5:** Comparison between different published methodologies for molecular detection of *T*. *cruzi* in açai samples.

Reference	Centrifugation prior DNA purification	DNA purification methodology	Exogenous Internal Control	DNA detection molecular-based methodology	qPCR efficiency	Validation with field samples
Ferreira et al., 2016 [30]	No	DNAzol (Invitrogen); NucleoSpin Food Kit (Machery-Nagel); CTAB	No	Conventional PCR Tc189Fw2/Tc189Rv3	-	No
Godoi et al., 2017 [24]	Triple centrifugation	Phenol-chloroform	No	Singleplex SYBR Green qPCR TCZ1/TCZ2; Ep1F/Ep1R	TCZ1/TCZ2: 80.82% Ep1F/Ep1R: 85.48%	No
Mattos et al., 2017 [22]	Yes	QIAamp DNA Mini Kit (Qiagen); QIAamp DNA Stool Mini Kit (Qiagen)	No	Singleplex TaqMan qPCR Cruzi 32/148 and TaqMan probe71 (FAM-NFQ)	Cruzi32/148: 91.25%	No
De Oliveira et al., 2019 [23]	Triple centrifugation	Phenol-chloroform	No	Conventional PCR TCZ1/TCZ2	-	No
Ferreira et al., 2018 [25]	No	CTAB	*rbcL* (plant chloroplast gene)	Conventional PCR Tc189Fw2/Tc189Rv3	-	Yes. Qualitative validation
Cardoso et al., 2020 [26]	No	Illustra Tissue and Cells genomicPrep Mini Spin Kit (GE Healthcare)	No	Singleplex SYBR Green qPCR TCZ3/TCZ4	TCZ3/TCZ4: 103.98%	No
Present study	Yes	High Pure PCR Template Preparation Kit (Roche Life Science)	Synthetic DNA (EXO-IPC, Applied Biosystems)	**Multiplex** TaqMan qPCR Cruzi1/Cruzi2 and TaqMan probe Cruzi3 (FAM-NFQ); EXO-IPC (Applied Biosystems)	Cruzi1/Cruzi2: 89.65%	Yes. Qualitative and quantitative validations

The first step of the present work was to verify if the methodology chosen for DNA extraction was efficient to extract a high-quality genomic DNA from *T*. *cruzi* in açai samples. One of the biggest challenges for DNA extraction from parasites in such a complex food matrix lies on the presence of inhibitors that can be co-purified with the DNA during the extraction step and can reduce the efficiency of the PCR. Açai is composed of a significant portion of lipids, proteins, carbohydrates, soluble and non-soluble dietary fibers, fatty acids, a variety of minerals, and high antioxidant compound content, like anthocyanins, and phenolic compounds [33]. Presently, there are several methods available for DNA extraction from different sorts of samples, food matrices included, and they can be based on commercial kits or can be in-house methods. Despite being low cost and offer a high concentration of DNA, in-house methods, such as phenol-chloroform and CTAB are often laborious and not suitable to assess large-scale analyzes, since they are not reproducible, simple, or rapid protocols. In relation to DNA extraction using a commercial kit, there is a concerning due to acai viscosity, which can impair the procedure [22], mainly because of clogging of the silica-membrane. Because of that, even though many commercial kits are available for extracting DNA from different matrices, only a limited number can be used for DNA purification from processed food products, which can make these kits even more expensive. Particularly, the kit used in this study has an inhibitor removal buffer that permits removing inhibitors residues that could have remained in açai samples.

As a first attempt to increase the sensitivity of the methodology in this study, artificially contaminated açai samples were submitted to a pre-lysis stage, through mixing an equal volume of açai and 2X of a lysis buffer (Guanidine-HCl 6M/EDTA 0.2M pH 8.0) at room temperature. Guanidine-HCl can disrupt cells in addition to inhibiting nucleases, being a chaotropic salt commonly used for isolation of nucleic acids from cellular extracts. Thus, this reagent facilitates the preservation of nucleic acids in biological fluids [34, 35] and the sample transport from the field to the laboratory. Several previous studies related to molecular diagnosis of *T*. *cruzi* in blood samples have already incorporated this step into the methodology, due to its importance as a stabilizing buffer to the sample [36–39].

The use of negative and positive controls in qPCR is essential to ensure the reliability of the reaction, avoiding false-positive or false-negative results. However, especially in qPCR assays it is also necessary to include an internal amplification control to monitor the reproducibility of DNA extraction and the absence of PCR inhibition (total or partial). In previous studies for the molecular diagnosis of Chagas disease, a target at the host DNA, such as RNAse P, or an exogenous DNA, were used as internal amplification controls, to avoid false negative results, which can occur when working with highly complex material [40], and to correct and normalize DNA variations between samples. However, to date, published studies related to *T*. *cruzi* molecular diagnosis in food samples, through qPCR, have not included an internal amplification control, despite using negative and positive controls in the reactions. In our study, we used a synthetic DNA to monitor the efficiency of the DNA extraction and the absence of inhibitors at qPCR. The reproducibility of the qPCR was confirmed since there was no variation in Exo-IPC Ct values, regardless *T*. *cruzi* concentration at the samples. Besides that, our internal amplification control was amplified in all açai samples, validating the true-negative results.

In most outbreaks, the presence of *T*. *cruzi* in food is, normally, detected by traditional methods, such parasite isolation and microscopic investigation. And, although this practice presents results with high specificity, microscopic examination is a labor-intense, time consuming method and has low sensitivity, being minimally effective when only a few microorganisms exist in a sample [41]. Namely, the characteristic dark color of the açai fruit, which is associated with high anthocyanin concentrations and a large amount of organic matter [33], greatly limit the possibility of parasites detection during microscopic visualization [42]. In this context, molecular methods can overcome these limitations and provide specific diagnosis and *T*. *cruzi* genotyping [25]. The qPCR developed in the present study showed improved sensitivity and linearity, that have also been observed in previous studies. Besides that, in the inclusivity assay, it was possible to observe that all the six *T*. *cruzi* DTUs could be detected in GEA samples. Despite the remarkable inclusivity of the multiplex real time PCR assay, we could also observe that the Dm28c (TcI) presented higher Ct values for the satDNA target than the other DTUs, in general. This result corresponds with previous observations that the *T*. *cruzi* DTU I present a lower number of copies for the nuclear satellite DNA [43]. Regarding the exclusivity assay, it was possible to observe that DNA of other trypanosomatids species showed no cross-amplification in any concentration tested. However, when we tested different concentrations of *T*. *rangeli* DNA, qPCR was positive, confirming the previous observed cross-amplification of *T*. *rangeli* with the selected primers and probe set [44, 45]. This expected result did not impair the use of this methodology to detect and quantify *T*. *cruzi* in açai samples, in cases of Chagas disease oral outbreaks, since *T*. *rangeli* will not cause any symptoms in humans, in contrast to the acute infection triggered by *T*. *cruzi*. In addition, there are important differences in the biological cycles of *T*. *rangeli* and *T*. *cruzi* inside the insect vector, where the former is practically not founded at the triatomine feces since it colonizes the salivary gland of the insects. On the other hand, it is expected that the açai contamination by *T*. *cruzi* occurs mostly due to triatomine feces contaminated with the parasite prior the insect itself. Thus, even in the presence of mixed contaminations *T*. *cruzi + T*. *rangeli* in triatomines at the açai palm tree, the presence of *T*. *rangeli* DNA in açai pulp should be rare. However, new molecular targets should be investigated to enhance the specificity for the *T*. *cruzi* parasite load quantification in açai samples.

In relation to the analysis of commercial açai-based samples, this work is, until now, the first one that has assessed, using a methodology based on Real-Time quantitative PCR, samples gathered from street markets of an endemic area. To build the standard curve to estimate the parasite load in the açai pulp samples, a T. cruzi clone from DTU I (TcI) was chosen, due to the previous identification of the high prevalence of TcI in samples from the Amazon region in Brazil [25, 46, 47]. The qPCR screening of these 45 GEA samples was shown to be more sensitive than microscopic examination, since the molecular method revealed a positivity of 20% (9/45), whereas all samples were negative for *T*. *cruzi* by direct observation at optical microscope. Probably it occurs due to the very low parasite load observed in most of samples, where 6 from the 9 positive samples presented parasite load below the reportable range (10^6^ to 1 par. Eq./mL), being detectable by the qPCR but not quantifiable with acceptable accuracy. Ferreira et al. [25] determined, in food samples commercialized in Rio de Janeiro and Pará states, *T*. *cruzi* contamination rates and molecular characterization through conventional PCR and multilocus PCR analysis, respectively. And, although the set of primers showed high specificity, since there was no amplification for *T*. *rangeli* and other trypanosomatids genomic DNA, the chosen target had lower sensitivity. Interestingly, from the 140 samples of açai-based products they analyzed, 14 samples (10%) were positive for *T*. *cruzi* DNA and triatomine DNA was also detected in one of these 14 samples. A previous study [48] also found insect fragments in açai samples and these findings may reinforce the link between açai and the presence of infected vectors or mammals near of the outbreak locations.

Regarding the use of molecular methodologies as a diagnosis tool, *T*. *cruzi* DNA detection by itself does not mean the presence of viable parasite in the sample, once DNA molecule can remain stable even a little after parasite death [23, 49, 50]. Nevertheless, the detection of the parasite DNA can be important to identify problems related to good manufacturing practices throughout the production chain and in the epidemiological investigation of orally acquired Chagas disease outbreaks. In this context, our results present a simple and rapid extraction protocol, directly from açai samples, with a simple step for sample stabilization and DNA extraction based on silica-membrane spin columns. In addition to a highly sensitive multiplex qPCR-based methodology, which includes a commercial exogenous internal positive control. The integrated methodologies standardized herein can assist in the surveillance of commercialized açai-based products, in a large-scale basis, and can be a powerful tool for a better understanding about orally acquired Chagas disease and for strategies to assure the safety of açai, such as in prevention, and control analysis. Of note, only the detection of *T*. *cruzi* DNA in açai pulp samples should not lead to the discarding of the food products, since this detection can remain positive even after the açai sterilization by blanching or pasteurization. Therefore, more studies should be performed to evaluate the potential of DNA and other molecules to detect infectant parasites in açai samples.

## Methods

### Ethics statement

The study was approved by the ethical committees of the *Universidade Federal do Amazonas* (CAAE: 97439918.5.1001.5020, Approval number: 2.961.307) following the principles expressed in the Declaration of Helsinki. Written informed consents were obtained from the owners of the street market stores.

### Açai samples

Forty-Five açai pulp samples, gathered randomly from different street markets in the Coari municipality (Amazonas States, Brazil) in June 2018, were used in this study. The açai pulps were purchased in a region comprised between 4°05'02.0"S 63°08'36.0"W at North, 4°06'04.0"S 63°08'49.0"W at South, 4°05'14.0"S 63°08'06.0"W at East and 4°06'01.0"S 63°09'02.0"W at West of the city. Prior the pulp preparation, açai fruits were submitted to sterilization at the stores, by blanching (the fruits are immersed in a water bath at 80°C for 10”, followed by the immersion in a water bath at room temperature for 2 minutes). Then, the pulps were prepared by the smashing of the açai fruit and mixing with water. All the açai fruits were collected at palms from the Coari municipality, except for 1 sample, collected at the Codajás municipality, also in the Amazon state.

### *Trypanosoma cruzi* cultivation

Strains and clones of *Trypanosoma cruzi*, belonging to subpopulations classified between DTUs I to VI: Dm28c (TcI), Y (TcII), INPA 3663 (TcIII), INPA 4167 (TcIV), LL014 (TcV) and CL (TcVI), were obtained from *Coleção de Protozoários* of *Fundação Oswaldo Cruz*, Rio de Janeiro, Brazil (Fiocruz, COLPROT, http://www.colprot.fiocruz.br). Epimastigotes were cultured in LIT (Liver Infusion Tryptose—BD, USA) medium supplemented with 10% of heat-inactivated Bovine Fetal Serum (Invitrogen, Massachusetts, USA), at 28°C for 5 days, to reach logarithmic growth phase. Parasites were harvested by centrifugation (3000x g for 10 minutes, at 4°C), washed three times with 0.15 M NaCl, 0.01 M phosphate buffer pH 7.2 (PBS) and resuspended in the same solution, prior to artificial contamination of açai pulp samples and DNA extraction. Parasite growth was estimated by counting cells in the Neubauer Chamber hemocytometer using an optical microscope and expressed as parasites/mL.

### Preparation of Guanidine-EDTA Açai (GEA) samples

For analytical validation, açai pulp samples were provided by the Instituto Nacional de Controle de Qualidade em Saúde (INCQS/FIOCRUZ), Rio de Janeiro, Brazil.

**Fig 3:**
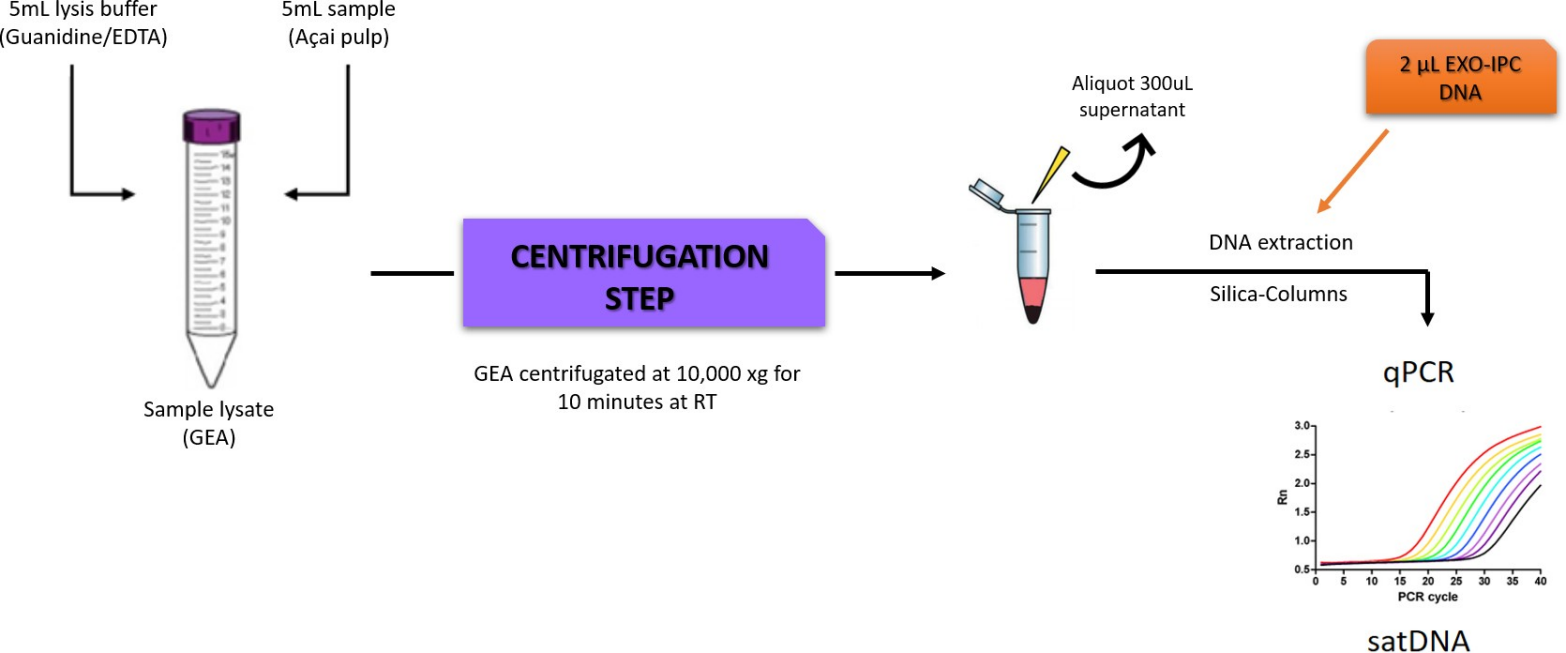
Scheme for the preparation of Guanidine-EDTA Açai (GEA) samples and DNA extraction using the Exogenous Internal Positive Control (Exo-IPC). The scheme shows the DNA extraction from the supernatant of the GEA spiked with EXO-IPC synthetic DNA, using a silica-column based commercial kit.

Samples were separated into 5mL aliquots and mixed with an equal volume of a lysis solution containing 6M Guanidine-HCL/0.2 N EDTA pH 8.0 (1:1 ratio). The total volume of açai lysate (GEA) was centrifugated at 10,000 xg for 10 minutes at room temperature. After the centrifugation, GEA supernatants were recovered and stored at 4°C until DNA extraction (Fig 3).

### DNA extraction

Each 300 μL of GEA supernatant sample was extracted using the High Pure PCR Template Preparation Kit (Roche Life Science, Mannheim, Germany), a kit based on silica-membrane spin columns technology. The DNA purification protocol was carried out according to the manufacturer's instructions, with some modifications, including the volume of sample that was increased from 200 to 300 μL. Briefly, during the proteinase K lysis step, the GEA samples were incubated for 2 hours at 56°C. In addition, at the DNA elution step, a volume of 100 μL of elution buffer was used to elute the purified DNA. The DNA samples were stored at -20°C and their purity and concentration were estimated using a NanoDrop 2000c spectrophotometer (Thermo Fisher Scientific, Massachusetts, USA) at 260/280 and 260/320 nm.

In the present study, to monitor the reproducibility of the DNA extraction and the absence of inhibitors at PCR, the commercial kit TaqMan Exogenous Internal Positive Control Reagents (Applied Biosystems, Foster City, CA, USA) was used. The EXO-IPC DNA is a synthetic molecule that presents no homology to any DNA sequences available in public databases. Thus, 300 μL GEA supernatant were spiked with 2 μL of the EXO-IPC DNA before DNA extraction. This EXO-IPC is supplied in a commercial kit format that also contains a set of pre-designed primers and TaqMan probe (VIC/TAMRA), targeting the synthetic DNA sequence.

For each DNA extraction batch (containing up to 11 samples), one negative control of the DNA extraction step was included, using a negative GEA supernatant sample. A sample was considered valid when the EXO-IPC target amplified with expected Ct values.

### Quantitative multiplex real-time PCR (qPCR) assays

Multiplex real time PCR assays were performed for the detection and absolute quantification of the Exo-IPC and *T*. *cruzi* satDNA targets in GEA samples, respectively. Reactions were carried out in a final volume of 20 μL, containing 5 μL of DNA as a template, 10μL of 2× FastStart TaqMan^®^ Probe Master Mix (Roche Life Science, Mannheim, Germany), 750 nM cruzi1 and cruzi2 primers and 250 nM cruzi3 probe (FAM/NFQ-MGB) targeting *T*. *cruzi* nuclear satellite DNA (satDNA) and 1μL of the 10× EXO-IPC Mix from the TaqMan Exogenous Internal Positive Control Reagents commercial kit (Applied Biosystems, Foster City, CA, USA), that contains a set of primers and probe (VIC/TAMRA) targeting the synthetic EXO-IPC DNA. Sequences of both sets of primers and probes are described in Table 6. Quantitative assays were performed on the Viia7 equipment (Applied Biosystems, Foster City, CA, USA) with the following cycling conditions: 50°C for 2 min, 95°C for 10 min, followed by 45 cycles at 95°C for 15 s and 58°C for 1 min. The threshold was set at 0.02 for both targets in all real time PCR assays.

**Table 6:** Primer sets and probes sequences for the multiplex qPCR assay.

Target	Primers/Probes	Sequences	Amplicon size	Reference
*T*. *cruzi* satellite DNA (Sat-DNA)	Cruzi 1 (Forward)	ASTCGGCTGATCGTTTTCGA	165 bp	Piron et al., 2007 [51]
	Cruzi 2 (Reverse)	AATTCCTCCAAGCAGCGGATA		
	Cruzi 3 (Probe)	FAM-CACACACTGGACACCAA-NFQ-MGB		
EXO-IPC (VIC/TAMRA)	Not Available	Not Available	Not Available	Applied Biosystems (4308323)

To build the standard curves for the *T*. *cruzi* absolute quantification, negative GEA samples were spiked with *T*. *cruzi* (Dm28c clone, TcI) to reach the 10^6^ epimastigotes/mL concentration, prior to supernatant isolation and DNA extraction. In parallel, DNAs were extracted from negative GEA sample supernatants and pooled, to be used as diluent for the standard curve. The curves were generated by ten-fold serial dilution of DNA from spiked GEA sample in DNA from negative GEA sample, ranging from 10^6^ to 1 *T*. *cruzi* equivalents/mL.

Each 96-well reaction plate included the standard curve, Negative Template Control (ultrapure water instead DNA template) and two positive controls (*T*. *cruzi* DNA at 10 fg/μL and 1 fg/μL.

### Inclusivity and exclusivity assays

For the development of qPCR methods some parameters for analytical validation were included, such as: Inclusivity study (i), comprising the detection of the target strains, and exclusivity study (ii), involving the lack of response of closely related non-target strains, which can be potentially cross reactive, but are not expected to be detected [52]. **(i) Inclusivity study:** to assess the ability of this qPCR methodology to detect the *T*. *cruzi* target, genomic DNA were tested from a representative panel of *T*. *cruzi* strains/clones belonging to the six different DTUs: Dm28c (TcI); Y (TcII); INPA 3663 (TcIII); INPA 4167 (TcIV); LL014 (TcV); CL Brener (TcVI). Samples were obtained from the serial dilution of DNA extracted from GEA spiked with 10^6^ parasites/mL, in negative GEA DNA, and assayed in duplicates, with concentrations ranging from 10^4^ to 10^−1^ parasite equivalents/mL. **(ii) Exclusivity study:** to assess this qPCR methodology’s lack of response from closely related non-target strains, serial dilutions of genomic DNA obtained from other species of trypanosomatids were tested: *Trypanosoma rangeli*, *Leishmania (Leishmania) amazonensis*, *Leishmania (Viannia) braziliensis*, *Herpetomonas muscarum*, and *Crithidia fasciculata*. Samples were assayed in duplicates, with concentrations ranging from 10^4^ to 0.1 parasite equivalents/mL.

### Optical microscopy

Açai pulp samples were analyzed for the presence of *T*. *cruzi* epimastigotes or trypomastigotes by optical microscopic examination. One drop from açai pulp was diluted in one drop of sterile saline solution, NaCl 0.85% (c.50 μL), on a glass slide and examined by microscopy between slide and coverslip, at magnifications of 200–400×.

### Statistical analysis

All experiments were performed at least in biological triplicates and experimental duplicates and data are reported as arithmetic mean ± standard deviation. All statistical tests were conducted using the SigmaPlot for Windows Version 12.0 (Systat Software, Inc., California, USA). Student’s t test or Mann-Whitney Rank-Sum tests were adopted to analyze the statistical significance of the apparent differences, according to the parametric or non-parametric distribution of the data. A p-value less than 0.05 was considered statistically significant (p<0.05).

The Tukey’s criterion (boxplots) [53] was used to detect samples with outlier Ct values for Exo-IPC (Cts>75th percentile+1.5×interquartile distance of median Ct), which would indicate inhibition or material loss in samples from a same experimental group with n>10”.

## Supporting information

S1 Table Concentration and purity (260/280 and 260/230nm ratios) of DNA extracted from açai samples purchased at Coari municipality (Amazonas States, Brazil). Açai samples were mixed with guanidine hydrochloride 6M-EDTA 0.2M pH 8.0 solution and centrifuged, as described. DNA was extracted from 300 μL of the supernatant.
(DOCX)

S1 File (ZIP)
