# Supplementary material for: Validation of a novel multiplex real-time PCR assay for Trypanosoma cruzi detection and quantification in açai pulp
Source: PLoS One. 2021 Feb 2;16(2):e0246435. doi: 10.1371/journal.pone.0246435 (PMC7853518; doi:10.1371/journal.pone.0246435)
Supplement: S1 Table — Açai samples were mixed with guanidine hydrochloride 6M-EDTA 0.2M pH 8.0 solution and centrifuged, as described. DNA was extracted from 300 μL of the supernatant. (DOCX) [file pone.0246435.s001.docx]

**S1 Table. Concentration and purity (260/280 and 260/230nm ratios) of DNA extracted from açai samples purchased at Coari municipality (Amazonas States, Brazil).** Açai samples were mixed with guanidine hydrochloride 6M-EDTA 0.2M pH 8.0 solution and centrifuged, as described. DNA was extracted from 300 µL of the supernatant.

| Samples | Concentration (ng/µL) | 260/280 nm ratio | 260/230 nm ratio |
| --- | --- | --- | --- |
| 1 | 347.1 | 1.8 | 1.1 |
| 2 | 66.4 | 1.8 | 2.2 |
| 3 | 92.3 | 1.8 | 1.4 |
| 4 | 113.7 | 1.8 | 2.1 |
| 5 | 53.1 | 1.8 | 2.2 |
| 6 | 51.0 | 1.8 | 2.4 |
| 7 | 45.8 | 1.7 | 2.2 |
| 8 | 53.4 | 1.7 | 2.0 |
| 9 | 63.0 | 1.8 | 2.5 |
| 10 | 78.4 | 1.8 | 2.1 |
| 11 | 88.9 | 1.9 | 2.0 |
| 12 | 58.7 | 1.7 | 1.7 |
| 13 | 172.0 | 1.5 | 0.9 |
| 14 | 184.3 | 1.6 | 0.8 |
| 15 | 130.6 | 2.0 | 2.2 |
| 16 | 107.2 | 1.8 | 1.9 |
| 17 | 118.0 | 1.9 | 2.2 |
| 18 | 98.5 | 1.9 | 2.3 |
| 19 | 110.4 | 1.9 | 2.2 |
| 20 | 110.5 | 1.9 | 2.2 |
| 21 | 130.3 | 1.9 | 2.2 |
| 22 | 124.6 | 1.9 | 1.9 |
| 23 | 50.1 | 1.8 | 2.9 |
| 24 | 183.4 | 1.6 | 1.0 |
| 25 | 63.7 | 1.8 | 2.1 |
| 26 | 52.1 | 1.8 | 2.3 |
| 27 | 49.5 | 1.8 | 2.2 |
| 28 | 48.3 | 1.8 | 2.4 |
| 29 | 68.1 | 1.8 | 2.2 |
| 30 | 74.2 | 1.8 | 2.3 |
| 31 | 59.7 | 1.7 | 2.3 |
| 32 | 52.3 | 1.7 | 1.7 |
| 33 | 57.9 | 1.7 | 1.8 |
| 34 | 58.7 | 1.9 | 3.0 |
| 35 | 36.9 | 1.6 | 1.7 |
| 36 | 73.6 | 1.9 | 2.6 |
| 37 | 53.2 | 1.8 | 1.8 |
| 38 | 51.5 | 1.8 | 2.6 |
| 39 | 49.9 | 1.8 | 1.8 |
| 40 | 90.2 | 1.8 | 1.7 |
| 41 | 48.5 | 1.9 | 2.7 |
| 42 | 59.6 | 1.8 | 3.0 |
| 43 | 62.9 | 1.8 | 1.6 |
| 44 | 74.0 | 1.8 | 1.9 |
| 45 | 60.7 | 1.9 | 2.6 |
